# Nuances in the control of separase activity between mitosis and meiosis

**DOI:** 10.1371/journal.pbio.3003206

**Published:** 2025-06-16

**Authors:** Martin Anger

**Affiliations:** 1 Institute of Animal Physiology and Genetics, Czech Academy of Sciences, Libechov, Czech Republic; 2 Department of Genetics and Reproductive Biotechnologies, Veterinary Research Institute, Brno, Czech Republic

## Abstract

Separase plays an important role in cleaving cohesin complexes that hold chromosomes together during cell division. This Primer explores a new study in PLOS Biology showing that control of separase activity is accomplished by cyclin B/CDK1 and securin, and does not involve SGO2 as reported in somatic cells.

## Teaser

Mammalian oocytes are prone to chromosome segregation errors, which frequently lead into aneuploidy and pregnancy loss. A new study in PLOS Biology addresses the role of the Mad2/SGO2 complex in the control of separase activity in these cells during meiosis.

From their emergence in the S phase, sister chromatids are held together by the cohesin complex [1]. The ties between sister chromatids are essential for accurate spindle assembly, as well as for the fidelity of chromosome segregation. Their final separation, during anaphase, requires the activity of a protease called separase, which cleaves kleisin subunits of cohesin complex, namely Scc1 in mitosis and Rec8 in meiosis [2]. This is an irreversible step, and when executed precociously, leads to aneuploidy, cell loss or tumorigenesis. In order to prevent premature activation of separase, and thus the cleavage of cohesin, several mechanisms are used by cells to control separase activity. Crucially, they are all linked to the completion of the assembly of the spindle apparatus. Therefore, only when all chromosomes, via their kinetochores, are correctly attached to the microtubules produced from spindle poles, is separase activation triggered [3].

Previous studies have identified two independent separase inhibitory mechanisms [4]. The first involves the interaction of separase with a specific protein called securin, which acts as its pseudosubstrate. The second is based on the phosphorylation of separase by cyclin B/CDK1 and its subsequent binding to this complex; however, this mechanism is only used in vertebrate cells [5]. The inhibition of separase by securin and cyclin B/CDK1 is mutually exclusive. The timing of separase inhibition is restricted by the duration of the activity of Spindle Assembly Checkpoint (SAC) and terminated by the onset of the activation of Anaphase Promoting Complex/Cyclosome (APC/C). Recently, a third mechanism was discovered in somatic cells, which involves separase inactivation by the Mad2/SGO2 complex, with SGO2 acting as a pseudosubstrate for separase, similar to securin [6]. Importantly, the inhibition of separase by the Mad2/SGO2 complex is at least partially linked to spindle assembly via the SAC and APC/C axis. In mitosis, all three mechanisms cooperate to prevent precocious separase activation, and when securin is depleted, the majority of separase is inhibited by the Mad2/SGO2 complex.

During meiosis I, the segregation of homologous chromosomes also depends on the cleavage of the cohesin complex by separase [7]. However, the important difference between meiosis I and meiosis II, and mitosis, is that separase cleavage on chromosome arms is limited to the cohesin located distally to the chiasmata. The remaining cohesin in the vicinity of the centromeres, which holds together sister chromatids, is protected from separase cleavage until anaphase II. As in mitosis, the activation of separase in meiosis I is postponed until completion of the spindle assembly, and the control of separase activity also involves securin and cyclin B/CDK1 inhibition [8]. Until now, there was no evidence of a role for the recently discovered Mad2/SGO2 complex in controlling separase activity in meiosis.

In their new study [9], Wetherall and colleagues tested this potentially important mechanism in mouse oocytes. The authors combined the depletion of SGO2 and securin proteins by morpholino, with simultaneous overexpression of separase resistant to cyclin B/CDK1 inhibition (separase S1121A) and monitoring of the separase activity in live oocytes using a localization-based cleavage sensor. Their results first confirmed that alone, either securin or cyclin B/CDK1 are sufficient for the control of separase activity in oocyte meiosis I, and for the proper timing of anaphase. When both control mechanisms were inactivated though, by simultaneous depletion of securin with morpholino and overexpression of separase S1121A, not only was separase activated earlier, but the oocytes also had substantial chromosome alignment and segregation defects, similar to those that occur with the simultaneous depletion of securin and SGO2 in somatic cells [6]. Interestingly, the simultaneous alleviation of both inhibitory pathways had only a small effect on the timing of the initiation of cohesin cleavage, and no effect on the timing of polar body extrusion.

The authors next focused on whether the separase activity in oocytes is controlled by the Mad2/SGO2 complex, similar to somatic cells [9]. They first stabilized SGO2 levels by mutating its KEN and D-box sites, which are recognized by APC/C. This procedure extended the stability of SGO2, but had no effect on polar body extrusion. Subsequently, they used morpholino oligos to reduce the SGO2 levels. This procedure alone, or in combination with simultaneous depletion of securin, and overexpression of separase S1121A, did not further exacerbate the effect they observed with the combination of securin depletion and separase S1121A overexpression.

**Fig 1:**
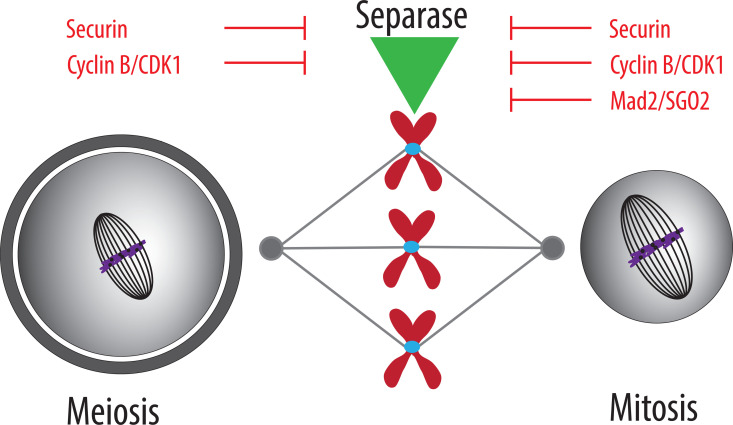
Differences in control of separase activity between meiosis and mitosis. Separase activity in mitosis is controlled by at least three different pathways: a specific protein inhibitor called securin; phosphorylation and binding to Cyclin/CDK1; and the Mad2/SGO2 complex. According to the results of the new study by Wetherall and colleagues [9], the Mad2/SGO2 complex does not play a role in separase control in oocytes.

The study by Wetherall and colleagues brings convincing evidence that, in contrast to mitosis, the Mad2/SGO2 complex is not required for the control of separase activity in oocyte meiosis I (Fig 1). Removal of securin and separase from the genome and replacement of separase with the S1121A mutant will, in the future, enable the analysis of chromosome alignment and segregation defects without residual amounts of proteins after morpholino depletion. The study also underlines the role of securin in the control of separase activity in these cells. Somatic cells seem to be more dependent on cyclin B/CDK1 to control separase, as the expression of a mutant resistant to CDK1 phosphorylation causes precocious separation of sister chromatids [6], whereas the oocytes are capable of controlling separase with securin alone. To understand why the Mad2/SGO2 complex is not essential for the control of separase activity in oocytes will require additional experiments. Whether this is due to the relatively high securin levels in oocytes, or due to the additional roles of Mad2 after SAC in somatic cells, remains to be determined. From that perspective, it would be interesting to assess the role of Mad2/SGO2 in developmentally related cells that have reduced securin levels, such as meiosis II oocytes or early cleavage cycle embryos.

Overall, the results of Wetherall and colleagues [9] demonstrate a significant difference in the control of separase activity between meiosis and mitosis. And considering the high frequency of chromosome segregation errors in female germ cells, the results contribute to our understanding of the etiology of aneuploidy, which is the most frequent reason for termination of development in mammals [10].

## Abbreviations

APC/C: Anaphase Promoting Complex/Cyclosome
SAC: Spindle Assembly Checkpoint
